# Dorsal hippocampus represents locations to avoid as well as locations to approach during approach-avoidance conflict

**DOI:** 10.1371/journal.pbio.3002954

**Published:** 2025-01-14

**Authors:** Olivia L. Calvin, Matthew T. Erickson, Cody J. Walters, A. David Redish

**Affiliations:** Department of Neuroscience, University of Minnesota, Minneapolis, Minnesota, United States of America; Center for Brain Research, Medical University of Vienna, AUSTRIA

## Abstract

Worrying about perceived threats is a hallmark of multiple psychological disorders including anxiety. This concern about future events is particularly important when an individual is faced with an approach-avoidance conflict. Potential goals to approach are known to be represented in the dorsal hippocampus during theta cycles. Similarly, important information that is distant from the animal’s position is represented during hippocampal high-synchrony events (HSEs), which coincide with sharp-wave ripples (SWRs). It is likely that potential future threats may be similarly represented. We examined how threats and rewards were represented within the hippocampus during approach-avoidance conflicts in rats faced with a predator-like robot guarding a food reward. We found decoding of the pseudo-predator’s location during HSEs when hesitating in the nest and during theta prior to retreating as the rats approached the pseudo-predator. After the first attack, we observed new place fields appearing at the location of the robot (not the location the rat was when attacked). The anxiolytic diazepam reduced anxiety-like behavior and altered hippocampal local field potentials (LFPs), including reducing SWRs, suggesting that one potential mechanism of diazepam’s actions may be through altered representations of imagined threat. These results suggest that hippocampal representation of potential threats could be an important mechanism that underlies worry and a potential target for anxiolytics.

## Abstract 2

Worry is a key component of anxiety, and is often characterized by imagination of a negative outcome. This study found that when a rat is attacked by a robot, hippocampal place fields develop to represent the robot. When the rat shows anxiety-like behaviors, it imagines the robot’s location, presumably worrying about the imminent danger.

## Introduction

Theories have long hypothesized that worry and anxiety involves imagination, specifically the ability to mentally simulate negative future outcomes. Indeed, the psychiatry literature has largely assumed this to be the case, positing that anxiety disorders result from cognitive schemas that distort one’s expectations and evaluations of the future [1–5]. Much theorizing has revolved around the notion of episodic future thinking in planning and goal-directed decision-making [6–8], but primarily in conditions of conflict between multiple positive outcomes (approach-approach conflict) [9,10]. In contrast, anxiety is thought to arise from conflict between motivations towards taking actions that one wants to both approach and avoid (approach-avoidance conflict) [11–14]. While theoretical work has long suggested a role for future thinking in these anxiety-inducing situations [4,5,15–17] and a specific role for planning-related structures such as hippocampus and prefrontal cortex therein [18], neurophysiological studies have concentrated on the emotional dimension of anxiety while largely ignoring the role of prospection [17,19–23].

Studies building on the spatial navigation literature have revealed that the hippocampus encodes mental simulations of hypothetical episodic scenarios during planning, encoding paths forward in both space and time to the next goal or subgoal [24–29]. Similarly, studies building on the same spatial navigation literature have revealed that the hippocampus plays out important information during behavioral pauses, including both appetitive [30–35] and aversive [36] memory recall. However, it remains unclear what role, if any, hippocampal fictive representations play in threat-based approach-avoidance conflict.

One such task is the predator-inhabited foraging task, an approach-avoidance conflict task that requires a rat to forage for food in the presence of a hostile robotic predator designed to mimic the hazardous environmental conditions faced by rodents in the wild [37]. The predator-inhabited foraging task (colloquially known as the “robogator” task) elicits a variety of anxiety-like behaviors in rats [37–39], including (1) choice-point hesitation at the nest entrance, which is similar to the stretch-attend posture, thought to arise from risk-assessment processes [40–43]; (2) mid-track retreat decisions, which are likely a kind of change-of-mind event [38,39]; and (3) slowing down during foraging, likely due to nervous concern about imminent dangers [38,44]. Consistent with this hypothesis, these behaviors are modulated by anti-anxiety drugs, particularly diazepam [39]. These behaviors depend on a complex neural circuit, including the amygdala [37,38], the bed nucleus of the stria terminalis [44], and the periaqueductal gray [45], as well as hippocampus [46]. Given the well-established role for hippocampus in prospective imagination of targets to approach, it becomes a particularly interesting question of how hippocampal representations encode the conflicting aspects of futures to approach and futures to avoid, and a particularly interesting question of how those drugs affect hippocampal processes.

The hippocampus shows 2 distinct states, identifiable by the oscillatory regimes revealed by the local field potential (LFP): a theta state, in which the LFP shows prominent 6 to 10 Hz (theta) and 15 to 25 Hz (beta) oscillations and a non-theta state (termed large-amplitude irregular activity or LIA) in which the LFP is dominated by 1 to 4 Hz delta band oscillations, punctuated by transient 150 to 250 Hz 150 ms events called sharp-wave ripples (SWRs) [47–52].

Hippocampal pyramidal cells are well known to encode contextual and spatial information key to episodic events within a task, particularly through the well-studied place fields that represent locations in spatial environments [47,48]. Each theta cycle entails a descending phase (as recorded from the hippocampal layer) in which pyramidal cell firing reflects the current location of the animal and an ascending phase in which pyramidal cell firing reflects the potential future paths of the animal [24,25,27,53–55]. On an avoidance task, Dvorak and colleagues [56] found transient decoding to the danger zone during that ascending phase of theta in which the locations that are typically decoded are the journey ahead of the animal [25,27,53,54,57]). However, it remains unknown how hippocampal activity reflects that conflict between approach and avoidance during theta cycles.

While in LIA states, the hippocampal pyramidal cell population bursts in transient high-synchrony events (HSEs) that reflect important information about the environment [29,31–35]. The specifics of the extent to which these transient events reflect planning [29,31,35,58], memory [31,32,35,59,60], learning [61–65], or some other process, such as development or maintenance of the spatial map [33,66–68] remains unknown. However, they have been observed to encode negative locations (shock-delivery locations) after novel experiences [36] and have been suggested to be keys to anxiety [69].

We sought to bring the anxiety, prospection, and ethology literatures together in order to interrogate the neural basis of negatively valenced episodic thinking. To do this, we recorded neural activity from dorsal hippocampal ensembles on an approach-avoidance task in which rats alternated between food locations, one of which was guarded by a robot pseudo-predator. We found hippocampal representational changes that created both sequential and discrete hippocampal representations of the threat that co-occurred with anxiety-like behaviors. We then found that diazepam had strong effects on the hippocampal processes that contained anxiety-related information during those anxiety-like behaviors. The findings reported below suggest that the dorsal hippocampus conveys both reward-related and threat-related information in approach-avoidance conflict environments and further suggest a role for the hippocampus in negatively valenced imagination, thus providing a novel neural mechanism that could underlie a long-hypothesized psychological key to anxiety.

## Results

**Fig 1:**
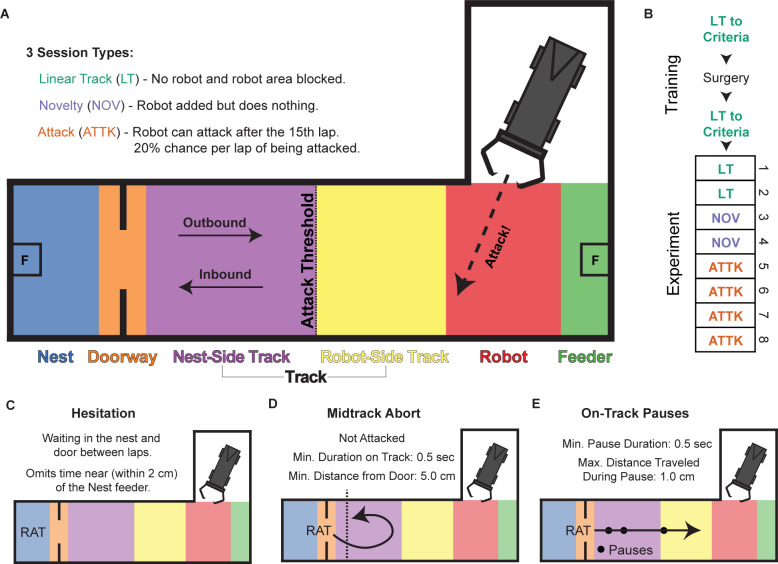
Task and experimental design. (**A**) The track was composed of a nest, track, and robot bay in the shape of an L. There were 2 feeders (marked as F). During Novelty and Attack sessions, a robot was placed in the robot bay, which was walled off during Linear Track sessions. During Attack sessions, there was a chance of the robot surging forth when the rat crossed the attack threshold. Based on these characteristics, we divided the linear portion of the track into Nest, Doorway, Nest-Side Track, Robot-Side Track, Robot, and Feeder segments. The separation between the “nest-side” and “robot-side” track segments was at the unmarked attack threshold. Outbound journeys entered from the Doorway zone and proceeded to the robot; Inbound journeys entered from the Robot zone and proceeded towards the nest. (**B**) The experiment consisted of 8 sessions: 2 Linear Track (LT), 2 Novelty (NOV), and 4 Attack (ATTK) sessions. Prior to the experiment, the rat was trained to alternate between feeders. (**C–E**) Putative worry behaviors that we examined. (**C**) Hesitation was defined as a period of waiting in the nest between laps. (**D**) MTAs were defined as a spontaneous retreat while approaching the robot that was not precipitated by the robot attacking. (**E**) On-track pauses were instances when the rat hesitated on the track. MTA, midtrack abort.

Neural ensembles were recorded from silicon probes bilaterally implanted into CA1 from rats (*n* = 6, 3M 3F) facing an approach-avoidance conflict in which food rewards at one end of a linear track were sometimes interrupted by the attack of a robot pseudo-predator. This conflict is similar to earlier versions of the robogator task [37,38,44], but increased the number of laps run in each session and required the rat to run past the robot to receive the food reward (Fig 1A), which allowed us to measure the spatial representations of near-robot locations. Rats received their entire daily complement of food during one 1 h session each day.

As pre-training, rats first learned to alternate ends of the arena in a linear track configuration (with the robot bay blocked off), receiving small (2× 45 mg) food rewards at each end of the track. Recordings were taken from an experimental sequence that consisted of 3 session types (Fig 1B). On Linear Track sessions (2 days), the robot was not present and the robot bay was walled off to create a purely linear track between the nest and far feeders. On Novelty sessions (2 days), the robot was present but did not move or make noise. On Attack sessions (4 days), the robot would occasionally (1 in 5 chance of attacking on each lap after the first 15 laps) screech, surge forward, and move its pincer-like jaws and stinger-like tail when the rat crossed an unmarked attack threshold as they moved from the nest to the far feeder. To assess habituation and learning, the Attack sessions were divided into 2 Early Attack sessions (first 2 of 4 days) and 2 Late Attack sessions (last 2 of 4 days), giving us 4 conditions to compare of two-sessions each. Sessions were analyzed in pairs for statistical power. For analytic purposes, we separated the track into spatial locations of the nest, doorway, nest-side track, robot-side track, the robot itself, and the far feeder, and we separated the rats’ movement on the track into outbound and inbound journeys that indicated when the rat entered the track while moving towards the feeder and the nest, respectively (Fig 1A).

Once the rats were attacked by the robot, we observed 3 identifiable behaviors that have often been seen in similar approach-avoidance conflict tasks and are putatively indicative of worrying about the robot [37–39,44,70]: hesitation in the nest, midtrack aborts (MTAs), and on track pausing. Hesitation in the nest entailed increased time spent in the nest space and thus reduced the number of robot approaches (Fig 1C). In addition to the time spent consuming the reward, time in the nest likely includes passive avoidance. Thus, increases in time spent in the nest likely indicates worry about being exposed to threat outside of the nest space. Midtrack aborts were instances when the rat left the nest into the track zone, but spontaneously returned before reaching the feeder on the other side of the robot (Fig 1D). Midtrack aborts likely indicate a change of mind from approach to avoidance behavior. Retreats from attacks were excluded from the identification of midtrack aborts as those retreats were a reaction to the robot attack rather than reflective of a proactive concern. Lastly, we noticed that the rats began to pause in their outbound journeys after being attacked (Fig 1E). This increase in on-track pauses was seen on outbound but not inbound journeys and likely indicates concern about the danger from the robot. Some of the rats changed their path of approach to the robot after being attacked (see S1 Fig). The modal change in approach was to avoid the robot more, but half of the animals either did not change their approach or angled more towards the robot.

### Hesitation in the nest

**Fig 2:**
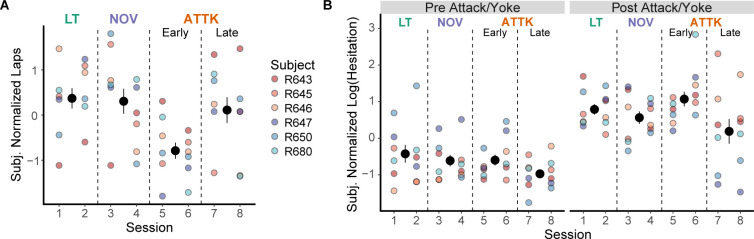
Effects of attack on hesitation behavior. (**A**) Laps run, normalized by subject. Note that for all figures, the large dots with error bars are the mean and standard error of the subjects’ average behavior and the small dots are the individual subject’s average values. The colors associated with each rat are indicative of the rat’s sex with shades of red indicating females and shades of blue indicating males. (**B**) Hesitation before and after being attacked, normalized by subject. For sessions that the rat was not attacked, we yoked the pre-post split time to the beginning of the lap number that the rat was attacked during the attack sessions. Underlying data and code can be found at https://osf.io/v6jt9/.

In approach-avoidance conflict, it is likely that a rat will be reluctant to approach the threat and may choose to avoid it entirely (Fig 1C). Rats ran fewer laps during the early Attack sessions (Fig 2A), and spent more time in the nest (Fig 2B). To differentiate early versus late behavior during each session, we split attack sessions (sessions 5 to 8) before versus after the first attack on that session. For non-attack sessions (sessions 1 to 4), we created a within-subject yoked match comparison by splitting time on the non-attack sessions based on the laps that the rats were attacked during the Attack sessions, which for simplicity we will term “pre/post” analyses (see Methods for additional details). This allowed us to compare (across sessions) durations on the maze that the rats had consumed similar amounts of pellets and had run similar numbers of laps. Rats had a tendency to remain in the nest longer in the second half of the session than the first half (presumably due to satiation); however, after being attacked, rats hesitated in the nest longer than on non-attack sessions. This change in hesitation and reduced lap running were only observed during early Attack sessions; by the late Attack sessions, the rats no longer hesitated for a prolonged duration (Fig 2B). Rats hesitated more before journeys towards the robot in the early Attack sessions after being attacked during that session compared to both the non-attack (Linear Track and Novelty) sessions and the late Attack sessions.

We statistically assessed the reduced number of laps run by the rats by fitting a linear mixed effects regression to the subject-normalized (in the statistical sense; see Methods) number of laps as a function of the session with a random effect of rat, and found a significant main effect of sessions, *F*(3, 39) = 4.81, *p* = 0.006. Rats ran significantly fewer laps on early Attack sessions than on Linear Track sessions, *t*(39) = −3.35, *p* < 0.01, and Novelty sessions, *t*(39) = −3.16, *p* = 0.02, and trended towards a difference when compared to late Attack sessions, *t*(38) = −2.60, *p* = 0.06. Rats did not run significantly fewer laps on late Attack sessions compared to Linear Track, *t*(39) = −0.75, *p* = 0.88, and Novelty sessions *t*(39) = −0.56, *p* = 0.94. Rats ran similar numbers of laps on Novelty and Linear Track sessions, *t*(39) = 0.19, *p* = 1.00.

Rat hesitation in the nest showed a similar pattern, which we assessed by fitting a linear mixed effects regression to the log-transformed hesitation. We found a significant interaction of session with pre/post, *F*(3, 2761.22) = 5.93, *p* < 0.001 and significant main effects of session, *F*(3, 2761.12) = 6.97, *p* < 0.001 and pre/post, *F*(1, 2762.89) = 330.15, *p* < 0.0001. Post hoc comparisons indicated that rats hesitated significantly longer during the post period (after the attack/yoke) across all sessions, all *t* scores >7.3, *p*s < 0.0001. During the pre-attack/yoke period, we did not detect significant differences in hesitation across the sessions, all |*t|* scores (2761) < 1.66, *p*s < 0.35. However, during the post-attack/yoke period, there were significant differences across the sessions with more hesitation during the early attack sessions (versus Linear Track: *t*(2761) = 3.60, *p* < 0.001, versus Novelty: *t*(2761) = 5.19, *p* < 0.0001, versus Late Attack: *t*(2761) = 6.31, *p* < 0.0001). We also detected that the rats hesitated less long during the Late Attack sessions than during the Linear Track sessions after being attacked, *t*(2761) = −2.85, *p* = 0.02, but there was not a significant difference when compared to the Novelty sessions, *t*(2761) = −1.29, *p* = 0.57.

These results suggest that early attacks by the robot caused greater hesitation, but that the rats habituated to it in the later Attack sessions. Overall, the rats showed an increase in hesitation at the nest after first being attacked, which led to a reduction in the number of laps that they ran when they began to be attacked, but, in both cases, they habituated to the threat by the later Attack sessions. In-nest hesitation may reflect the safety of the nest and active avoidance of the on-track threat.

#### Threat caused shift in spatial tuning of hippocampal cells

Place fields are thought to encode episodic memories [48,71] and have been observed to change in response to those events, encoding the location of the animal at the time of those events [72–74]. While previous work has found changes to spatial tuning in response to attack by the robot on similar tasks, in those previous task versions, the robot attacked towards the food pellet and the analyses could not differentiate the location the animal was at when attacked from the location of the robot itself or that of the reward site [46]. Previous work on the version of the task used here (linear track with a guarding robot attacking from the side) found a trend that spatial tuning was more stable on the nest side than the robot side [2].

**Fig 3:**
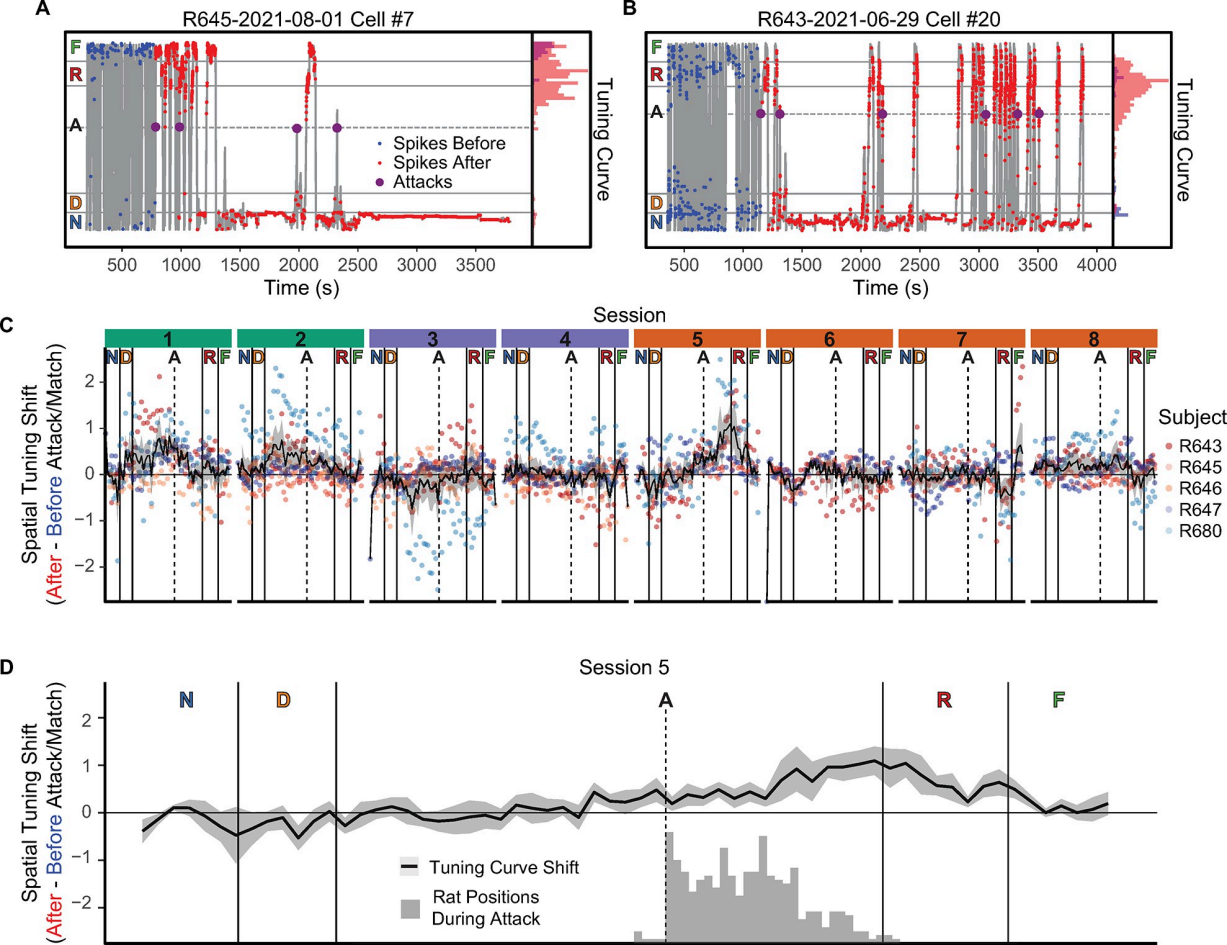
Effects of attack on spatial tuning. (**A, B**) Example cells that developed place fields between the point of attack and the robot after being attacked the first time. The histograms on the right panels show the occupancy-normalized tuning curves. (**C**) The change in spatial tuning (i.e., the average change in cell occupancy-normalized tuning curves) before and after being attacked. The black line with gray fill indicates the average spatial tuning at each position and the standard error, and the small dots are each subjects’ shift at each tuning-curve spatial bin on the maze. Values above 0 indicate more spatial tuning to the location after the attack and values below 0 indicate less. Note the shift in tuning on the first attack session (session 5) after being attacked around the location of the robot. (**D**) The position of the rat 0 to 0.5 after the attack was initiated compared with the spatial tuning shift. Underlying data and code can be found at https://osf.io/v6jt9/.

The first attack by the robot produced a notable change in the spatial tuning of hippocampal place cells. These changes occurred in representations of space beyond the location of the animal when attacked, and instead appeared at the location of the robot and the approach to it (which was not where the animal was when attacked). Fig 3A and 3B show 2 examples of this shift in which the cell’s place fields changed to encode the space between the attack threshold and the robot.

To quantify how spatial tuning adjusted to reflect the robot’s location, we determined the occupancy-normalized tuning curves based on each cell’s spiking activity prior to and after the first attack, and calculated the difference to determine the occupancy-normalized spatial shift for each cell. After calculating this by cell, we averaged across the cells to establish the average change in spatial tuning at each location (Fig 3C). For sessions during which the rat was not attacked, we used the yoked laps as defined above. On the first attack day (session 5), there was a marked shift in the spatial tuning after the first attack in the robot and robot-side track spatial zones.

We statistically assessed the shifts in spatial tuning by running a general linear model of the cell tuning curves as a function of the session and track zone. The GLM indicated that for the tuning curves on the approach to the robot there was a significant interaction of session and zone, *F*(35, 4189) = 7.16, *p* < 0.0001, a main effect of zone, *F*(5, 4189) = 6.83, *p* < 0.0001 and a main effect of session, *F*(7, 4189) = 12.85, *p* < 0.0001. Tukey post hoc comparisons indicated that there was a greater shift of spatial tuning in the robot-side track and robot zones on the first attack session (session 5) than on other sessions (robot-side track zone: |*t*| scores >5.42, *p*s < 0.0001; robot: all |*t*| scores >3.27, *p*s < 0.02). There was, also, significantly more spatial tuning to the nest-side track zone during the yoked post-attack period when the rats were running the Linear Track sessions than other sessions, all |*t*|s>3.98, *p* < 0.01. These results suggest that the attack by the robot caused the spatial tuning to shift to the robot and the approach to it.

Importantly, the change in the spatial tuning was not directly reflective of the position of the rat during the attack (Fig 3D). While the positions of the rat during the 0 to 0.5 s after the attack was initiated were past the attack threshold, they did not reflect the largest change in spatial tuning, which was at the boundary of the track and robot sections of the linear track.

Overall, these results suggest that the rat’s hippocampus increased spatial tuning to the robot, its location, and the approach to that location after it was attacked the first time. Importantly, unlike previous observations of place field appearances [72–74], this increased tuning encoded locations of the robot and the final approach to it rather than the location of the rat when the event occurred.

#### Threat decoding during hesitation

As discussed in the introduction, theories suggest that worry entails representations of potential threats [1–4]. Given that dorsal hippocampal activity can encode information about other places and other times [48,55,59,60,75,76], particularly in conditions of concern [36,56], we assessed the effect of the changes in hippocampal spatial tuning on the transient representations during HSEs during the increased hesitations after attack. If HSEs are used to replay information and there was a need to represent a salient event, then the rat’s hippocampus could increase the number of HSEs or the tuning of each HSE to the threat during the event.

**Fig 4:**
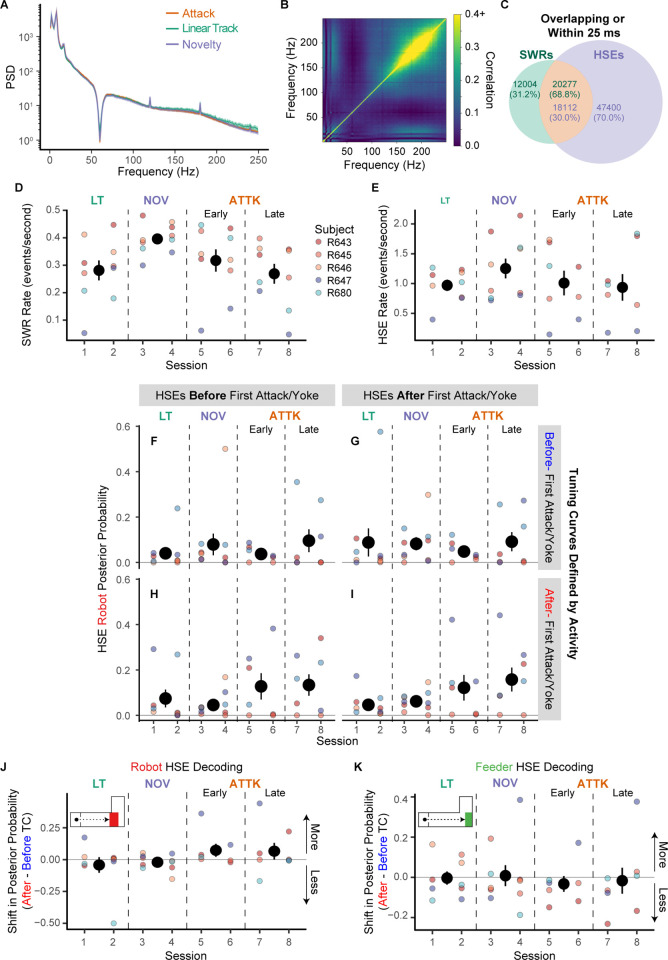
Changes in decoding during SWRs and HSEs. (**A**) The average power spectral densities while rats were in the nest across sessions. A bandstop filter was used to reduce the effect of 60 Hz AC line noise. (**B**) The average cross-frequency correlation, which indicates significant SWRs (150–250 Hz). (**C**) The overlaps in SWRs and HSEs. The numbers indicate the event counts and, in parentheses, the percentages. The different colors indicate whether the overlap is determined based on the SWRs (green) or HSEs (purple). (**D**) The SWR rate while the rats were in the nest. (**E**) The HSE rate while the rats were in the nest. (**F–I**) The posterior probability of the robot’s location. The panels are separated by whether the HSEs were before (pre-) or after (post-) the first attack/yoked lap and whether the decoding was performed based on tuning curves from before or after the first attack/yoked lap. These panels suggest that the shift in cell tuning curves mediates the increased decoding of the robot’s location while rats hesitated in the nest. (**J**) The difference in posterior probabilities of the robot’s location using before- and after-attack tuning curves. (**K**) The difference in posterior probabilities of the feeder location using pre- and post-tuning curves. Underlying data and code can be found at https://osf.io/v6jt9/. HSE, high-synchrony event; SWR, sharp-wave ripple.

During the time spent hesitating in the nest and at the nest entrance, CA1 LFPs had low theta power, instead showing increased delta (1 to 4 Hz) and SWR (150 to 250 Hz) power, indicative of large-amplitude irregular activity (Fig 4A; LIA, [47–49]). SWRs were also indicated by cross-frequency correlations, which highlight a block of highly correlated frequency power in the SWR band (Fig 4B). We decoded during HSEs, which overlaps with most SWRs (Fig 4C). We did not see a marked increase in the rate of SWRs (Fig 4D) or HSEs (Fig 4E; *F*(3,11) = 2.49, *p* = 0.11) while the rats were in the nest after that first attack. We did detect a main effect of session with the SWR rate, *F*(3,12) = 3.66, *p* = 0.04, but none of the post hoc comparisons survived correction for multiple comparisons, all |*t*|-scores <2.88, *p*s > 0.05.

Importantly, the content encoded in the neural ensembles could have also changed. Earlier work indicated an increase in decoding to the robot location during SWR events during hesitation in the nest after the rat had been attacked [2]. However, these analyses used tuning curves derived from the entire session. If tuning curves change, this could produce a change in the decoded posterior of HSEs. Greater decoding to a location could be due to multiple factors. An increased decoding to the robot’s location could arise either from a change in which cells participate in the HSE (due, for example, to what aspects of the world are attended to during those HSEs), or by a change in the underlying representation of the world (due, for example, to a change in the place fields).

To determine what was causing the increased decoding of the robot’s location during HSEs, we compared 4 conditions: decoding of HSEs occurring before and after the first attack (or yoked lap) crossed against spatial tuning derived from experiences occurring before and after the first attack (or yoked lap) (Fig 4F–4I). If there is a change in the spatial tuning of the neurons, then the neural decodings of the pre-attack/yoke period that utilize the post-attack/yoke tuning curves should have an increased posterior probability at the robot’s location. Whereas, if the increased decoding to the robot’s location is driven by which neurons spike during the HSEs, then the neural decodings of the post-attack/yoke period that utilize the pre-attack/yoke tuning curves will still exhibit the increased decoding to the robot’s location. We observed the former. There was more decoding to the robot’s location when we used the tuning curves that were defined by activity post-attack/yoke (Fig 4H and 4I), but not when we used the pre-attack/yoke (Fig 4F and 4G), even for HSEs that occurred before the rat was attacked (Fig 4H). This suggests that the increased decoding to the robot’s location during HSE’s was mediated by shifts in spatial tuning rather than which cells were active during the HSEs.

We statistically assessed this shift by fitting a general linear mixed effects regression to the posterior probability of the robot during high synchrony events occurring in the post-attack/yoke period as a function of whether the rat had been attacked that session or not with a random factor of rat to control for individual differences. The general linear mixed effects regression detected a significant interaction, χ^2^(3) = 946.43, *p* < 0.0001 and main effect of session, χ^2^(3) = 365.40, *p* < 0.0001, but no main effect of whether we used the pre- or post-tuning curves, χ^2^(1) = 0.00, *p* = 0.997. Post hoc comparisons indicated that when using the after-attack tuning curves, there was more decoding of the robot during the early and late Attack sessions than the Linear Track and Novelty sessions (early Attack to Linear Track: *z* = 15.94, *p* < 0.0001, early Attack to Novelty: *z* = 7.16, *p* < 0.0001, late Attack to Linear Track: *z* = 27.17, *p* < 0.0001, late Attack to Novelty: *z* = 22.45, *p* < 0.0001). There was also significantly less decoding of the robot location on Linear Track than on Novelty sessions, *z* = −11.76, *p* < 0.0001. When using the pre-attack/yoke tuning curves, we no longer detected this relationship. With pre-attack/yoke tuning curves, there was no detectable difference between the decoding during early Attack and the Linear Track sessions, *z* = 1.96, *p* = 0.20 and less decoding to the robot’s location than during Novelty sessions, *z* = −11.11, *p* < 0.0001. Similarly, with pre-attack/yoke tuning curves, there was less decoding of the robot during late Attack sessions than during the Linear Track, *z* = −4.45, *p* = 0.0001, and Novelty sessions, *z* = −17.31, *p* < 0.0001.

For comparison, we assessed decoding of the feeder location during these same HSEs and found the opposite pattern (differences summarized in Fig 4K). When using the post-attack tuning curves, there was significantly less decoding of the feeder location during early and late Attack sessions than on Linear Track and Novelty sessions (early Attack versus Linear Track: *z* = −7.79, *p* < 0.0001, early Attack versus Novelty: *z* = −3.00, *p* = 0.01, late Attack versus Linear Track: *z* = −8.52, *p* < 0.0001, late Attack to Novelty: *z* = −3.30, *p* < 0.01). When using the pre-attack tuning curves, there was more decoding of the feeder location during early and late Attack sessions than on Linear Track and Novelty Sessions (early Attack versus Linear Track: *z* = 15.99, *p* < 0.0001, early Attack versus Novelty: *z* = 11.33, *p* < 0.0001, late Attack versus Linear Track: *z* = 19.89, *p* < 0.0001, late Attack versus Novelty: *z* = 15.57, *p* < 0.0001).

These results indicate that there was increased decoding to the robot’s location during HSEs occurring while the rat was in LIA, hesitating in the nest, after the animal was attacked, but that this increased decoding did not arise from an increased probability of events being released, nor from a change in the set of cells firing during the event. Instead this increased decoding came from changes in place field encoding. The hippocampal spatial tuning had shifted to more highly represent the location of the robot and thus HSEs were more likely to pull up that representation. Hippocampal location-specific discharge had changed to signal location of the robot more often, and thus the mental explorations of the world during HSEs more often included the vicinity of the robot.

### Midtrack aborts

**Fig 5:**
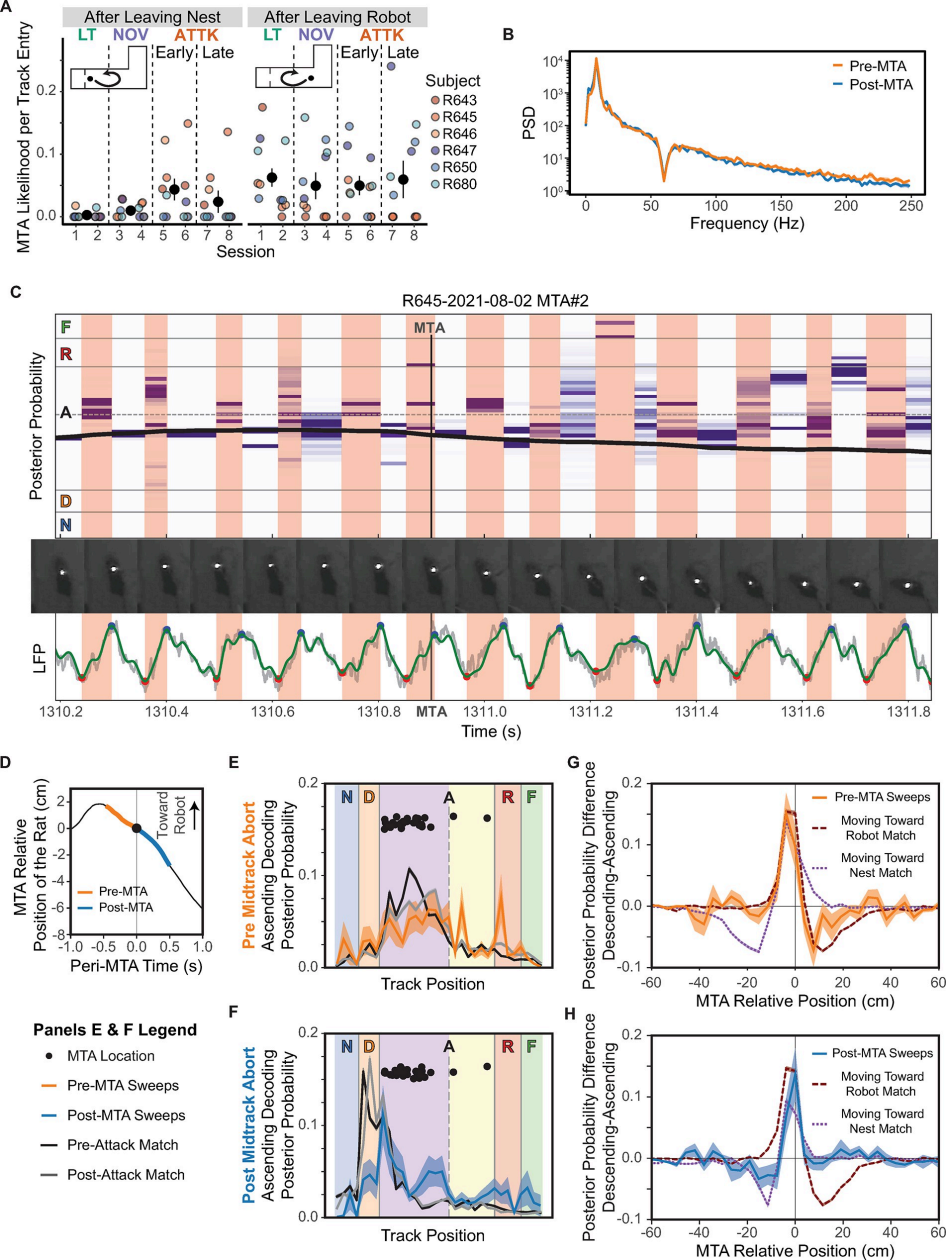
MTAs. (**A**) Probability of an MTA per entry onto the track. (**B**) The PSD before and after an MTA. (**C**) Example of how the decoded posterior probability (shown from low-to-high probability as white-to-purple) changes around the midtrack abort. The animal’s position is indicated by the black line. The video frames below the decoding highlight the rat’s movement around the identified MTA. The white dot on the rat is the illumination from an LED, which is how we tracked the rat’s position. The LFP shows the identified theta peaks (blue) and troughs (red), which provide the ascending (red background) and descending (white background) phases of theta. The green line shows the 6–40 Hz bandpass filtered LFP. (**D**) The average position of the rats around a midtrack abort (black), with the pre-attack and post-attack periods of investigation respectively highlighted orange and blue. (**E**) The posterior probability during the ascending phase of theta before the midtrack abort. (**F**) The posterior probability during the ascending phase of theta after the midtrack abort. (**G**) The direction of the decodings from the descending to ascending phases of theta before the midtrack abort. (**H**) The direction of the decodings from the descending to ascending phases of theta after the midtrack abort was initiated. Underlying data and code can be found at https://osf.io/v6jt9/. LFP, local field potential; MTA, midtrack abort; PSD, power spectral density.

During an approach-avoidance conflict, an individual may initially decide to approach a reward, but then be overwhelmed by the potential threat as it becomes more salient and switch to an avoidance strategy. On this task, this change of strategy manifests as a midtrack abort [37–39,44] (Fig 1D). Consistent with this previous work, after being attacked, rats began to engage in more midtrack aborts when approaching the robot (Fig 5A). We did not see a similar change in the number of reorientations on journeys back to the nest (i.e., starting from the feeder near the robot and returning back to that feeder without reaching the nest first; Fig 5A), implying that the increase in these outbound midtrack aborts were selective to the approach-avoidance conflict that occurs only on the outbound journey towards the robot, and not to other factors such as exploration or confusion, which would be expected to increase both outbound and return mid-track aborts.

We statistically assessed this spontaneous switch from approach to avoidance behavior by fitting a binomial regression to whether the animal aborted during a journey as a function of session and direction of travel. This analysis found that there was an interaction of direction of travel and session, χ^2^(3) = 21.87, *p* < 0.001, a main effect of session, χ^2^(3) = 16.87, *p* < 0.001, and a main effect of direction, χ^2^(1) = 21.87, *p* < 0.001. When approaching the robot after leaving the nest, the rats made significantly more midtrack aborts on early Attack sessions than during Linear Track sessions, *z* = 3.75, *p* = 0.001 and Novelty sessions, *z* = 3.52, *p* = 0.002. This was somewhat attenuated during the late Attack sessions. The rats were not significantly less likely to engage in a midtrack abort in late versus early Attack sessions, *z* = 1.94, *p* = 0.21, but were more likely to spontaneously abort an approach in late Attack versus Linear Track sessions, *z* = 2.75, *p* = 0.03. However, we did not detect a significant difference in how likely the rats were to perform midtrack aborts between the Linear Track and Novelty sessions, *z* = 1.80, *p* = 0.27. As expected, the rats did not significantly differ in the number of midtrack aborts during Linear Track and Novelty sessions, *z* = −1.54, *p* = 0.41. There were no significant differences in the number of midtrack aborts across the sessions when returning to the nest (all |*z*| scores <= 1.5, *p*s > 0.43).

Overall, the rats showed an increase in the number of midtrack aborts on outbound journeys. This increase was selective to the approach-avoidance conflict as indicated by the increase only occurring when approaching the robot on outbound journeys. A subset of rats also turned around before completing the inbound journey, but these inbound behaviors were insensitive to the presence of the robot. Importantly, the robot only attacked rats on outbound journeys, and thus inbound journeys were not dangerous. The causes of these inbound behaviors, which revealed strong individual differences, but did not change after being attacked, remains unclear.

#### CA1 activity encoded the attack location during midtrack aborts

Extensive previous data has found that as rats are running towards a goal, the dorsal hippocampus shows theta oscillations [47,52]. The descending phase of the theta cycle (as recorded from the hippocampal pyramidal layer) encodes the location of the animal and the ascending phase encodes potential future paths, forward towards the next goal [9,24–28,53,57]. If hippocampal information of a threat’s location is being used to determine whether the animal should retreat as it approaches, then that information will most likely be observed during the ascending phase of theta oscillations. Fig 5C shows an example of how the decoding changed around the time of a midtrack abort. In this example, the decoding posterior probability during the descending component of theta (shaded white) encoded the location of the animal quite accurately (black line). In contrast, during the ascending component of theta (shaded red), the decoding posterior probability was concentrated substantially ahead of the animal’s position prior to the MTA. This you-are-here/future-paths structure is consistent with theta sequences seen in goal-approach (non-threat-based) tasks [25,27,53,77–80].

However, after initiating the MTA, these local (descending) and forward (ascending) posteriors during theta became more varied. There were no apparent changes in the low-frequency power spectral density (PSD) before versus after the MTA (Fig 5B; no significant differences in 1 to 4 Hz delta, *t*(41) = −2.37, *p* = 0.02, 6 to 10 Hz theta, *t*(41) = 0.57, *p* = 0.57, or 15 to 20 Hz beta, *t*(41) = −1.43, *p* = 0.16, after a family-wise error correction), although there was a significant decrease in 120 to 250 Hz SWR power band power, *t*(41) = 3.03, *p* = 0.004. Given the observed PSD, this SWR-range significant difference may have been due to changes in the aperiodic component rather than oscillatory activity [81], as no SWR events were observed. These analyses suggest that the hippocampus remained in the theta state during midtrack aborts.

To determine the relationship between the hippocampal activity and the behavior around the midtrack abort, we decoded spatial posteriors during the ascending theta phase 0.5 s before and after the MTA event (Fig 5D). We aligned the data to the moment when the rat’s head began to turn into the retreat, which typically occurred after the rat had already stopped their forward momentum. We compared the decoding during the ascending phase of theta (usually forward of the animal) around the MTA to when the animal was in the same position and moving in the same direction. We separated our comparisons by whether the animal had been attacked yet in a given session since that affected the rats’ movement rates and, likely, their action planning. The ascending theta decodings around the MTAs were atypical to matched comparisons (Fig 5E and 5F). Prior to the MTA, we observed more decoding of the robot and the attack threshold than matched comparisons (Fig 5E). The decoded posteriors prior to the MTA tended to be more varied than matched comparisons. Importantly, the ascending theta decodings we observed after the MTA were unlike their matched counterparts (Fig 5F). The ascending theta phase decodings in the 0.5 s after the MTA became more like when moving towards the nest, but still included greater decodings of the robot’s location and the attack threshold, both of which were behind the rat (Fig 5F).

Since an MTA is a switch in the rats’ direction of travel, the local-to-ahead direction of the theta decoding should track the anticipated action. We assessed this by comparing the 5 theta cycles before and after the MTA with position and direction matched controls. By subtracting the decoding during the “local” descending phase of theta from the “forward” ascending phase, we could assess how much of the decoded posterior was local and how much included regions ahead or behind the animal across the theta cycle (Fig 5G). We assessed this by decoding the descending (shaded white in Fig 5C) and ascending portions (shaded red in Fig 5C) separately and subtracting them (descending - ascending). This typically shows a clear distinction between the 2 decodings [27,82]. Despite the rat being stationary, the second half of theta had significant forward decoding towards the outbound journey (Fig 5G). These decoded probabilities were more attenuated than matched passes, which could be due to the sequences only going to the attack threshold or the robot location rather than all the way to the far feeder (Fig 5E). Similarly, once the rat had started their retreat, the direction of the theta decoding tracked the rat’s future direction of travel back to the nest (Fig 5H), even though the rat had not fully turned to face that direction (see, for example, Fig 5C). Importantly, however, the ascending components were attenuated forward (toward the nest) and included components behind the rat (where the robot was; Fig 5H), suggesting an attention to dangers even behind the rat during these theta states.

### On-track pauses

**Fig 6:**
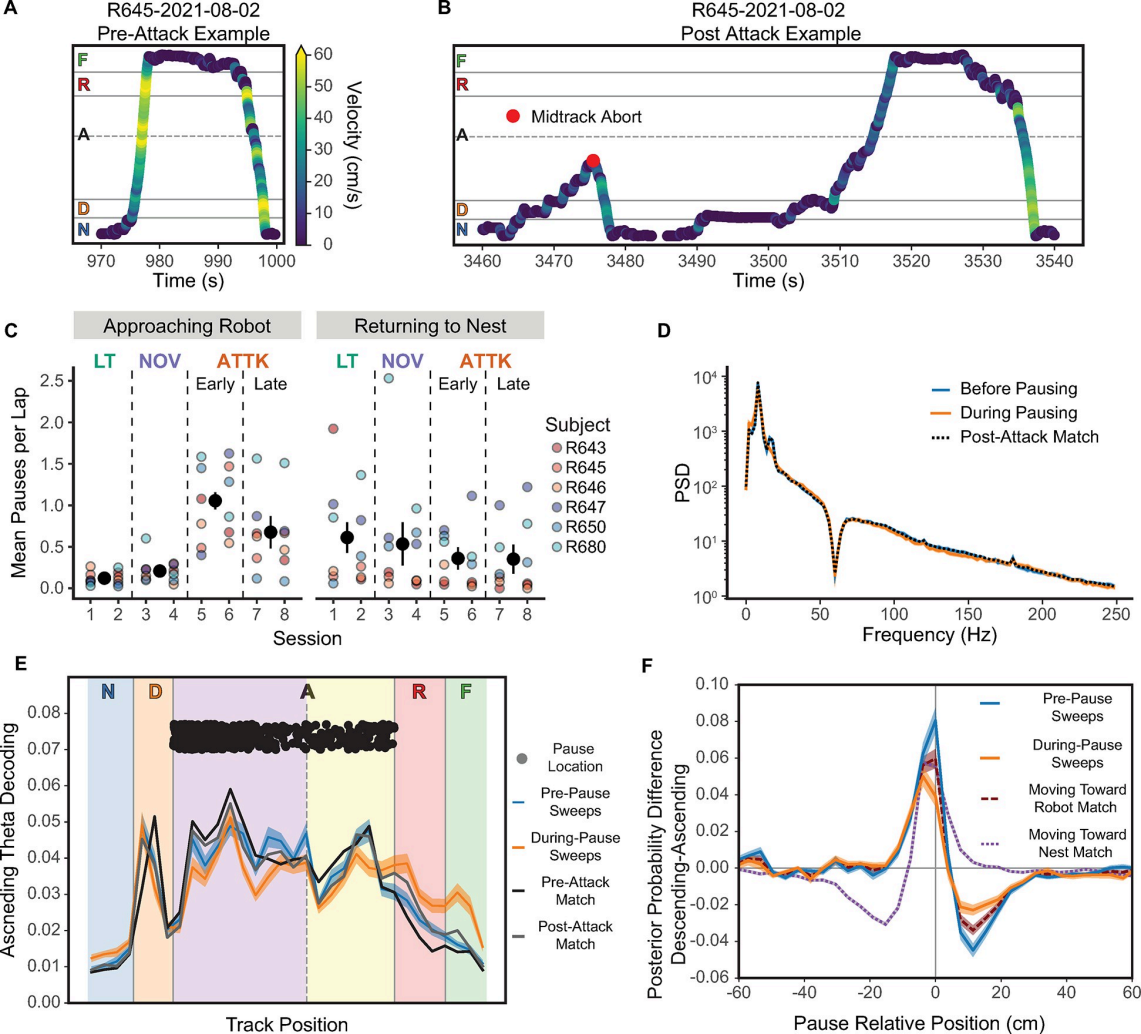
On-track pausing. (**A**) Example of rapid movement on a lap prior to being attacked (the first attack on this session was at 1,091 s). (**B**) Example movement of the same rat that now shows substantial pausing on the track after being attacked. (**C**) The average number of pauses per lap during the approach to the robot. (**D**) The PSD prior to and after the pause was initiated. (**E**) Decoded posterior position during the ascending phase of theta prior to pausing on the track. (**F**) The difference between descending and ascending theta phase decoding, prior to and during pausing. Underlying data and code can be found at https://osf.io/v6jt9/. PSD, power spectral density.

We observed that the rats began to repeatedly pause as they moved towards the robot after it had attacked them (Fig 1D, compare Fig 6A with Fig 6B). We observed a marked increase in the number of pauses on outbound journeys when the rat was approaching the robot after being attacked (Fig 6C). During non-attack sessions, the rats rarely paused when approaching the feeder, but this on-track pausing behavior increased dramatically after the rat was attacked. There was not a similar decrease of on-track pausing on return journeys, suggesting that these pauses were related to danger of approaching the robot.

We quantified these changes by fitting a zero-inflated Poisson generalized linear mixed effects regression to the number of pauses on a journey as a function of the session and the direction of travel with a random factor of rat. We found a significant interaction of the session and direction of travel, χ^2^(3) = 316.54, *p* < 0.0001, and main effects of session, χ^2^(3) = 202.81, *p* < 0.0001, and direction of travel, χ^2^(1) = 32.64, *p* < 0.0001. Rats paused significantly more often on the outbound journey during the early and late Attack sessions compared to the Linear Track sessions (versus early Attack: *z* = 17.97, *p* < 0.0001; versus late Attack: *z* = 14.47, *p* < 0.0001) and Novelty sessions (versus early Attack: *z* = 15.32, *p* < 0.0001; versus late Attack: *z* = 11.11, *p* < 0.0001). We found increased pausing on the early Attack sessions compared to the late Attack sessions, *z* = 5.83, *p* < 0.0001, consistent with habituation. Rats also paused less on Novelty sessions than on Linear Track sessions, *z* = −4.28, *p* = 0.0001, suggesting that the pauses were due to being attacked, and not simply to the presence of the robot. We did not detect any significant differences in the rate of pausing during the return journeys, all |*z*|s < 2.49, *p*s > 0.06.

Rats began to show these on-track pauses only after being attacked by the robot and not simply by its presence—on-track pausing did not increase during the Novelty sessions, but only during the early Attack sessions. Rats continued to show on-track pausing during all 4 Attack sessions, but did show some habituation between the early and late Attack sessions.

#### CA1 remained in theta during on-track pausing

If the on-track pauses are considerations of potential future danger (like MTAs), we would expect CA1 to remain in theta and show representations of the robot. If the on-track pauses are moments of hesitation, we would expect CA1 to be in LIA and show SWRs and HSEs that represent the robot. While we did find a decrease in 6 to 10 Hz theta power (*t*(609) = −7.12, *p* < 0.0001), an increase in delta power (*t*(609) = 5.48, *p* < 0.0001), and a major decrease in 15 to 20 Hz beta power (*t*(609) = −17.55, *p* < 0.0001), theta power remained relatively strong throughout the pause (compare Fig 6D to Fig 4A). We also saw a significant decrease in SWR power during these pauses (*t*(609) = −5.62, *p* < 0.0001) and a significant decrease in HSE count (*t*(14) = 3.49, *p* = 0.004), strongly suggesting that CA1 remained in relatively normal theta through the pause.

We also found some evidence for increased CA1 representation of potential threats and rewards during on-track pauses (Fig 6E). During pauses, neural activity in the ascending theta component decoded to the robot and feeder locations more than matched comparisons or the pre-pause activity. To determine whether anything in the hippocampal activity might have initiated the on-track pause, we assessed the decoding during the ascending (“forward”) component of theta (Fig 6F). There tended to be a larger difference between the decoded posteriors in the descending and ascending phases prior to pause than during the pause. These data suggest that pauses are somewhat similar to MTAs in that there is greater representation of the robot’s location. Importantly, the increase in feeder location representation during the pause (Fig 6E) as compared to pre-MTA theta cycles (Fig 5E) may be what differentiates the 2 actions (continuing forward versus retreating).

### Pharmacological manipulations with diazepam

**Fig 7:**
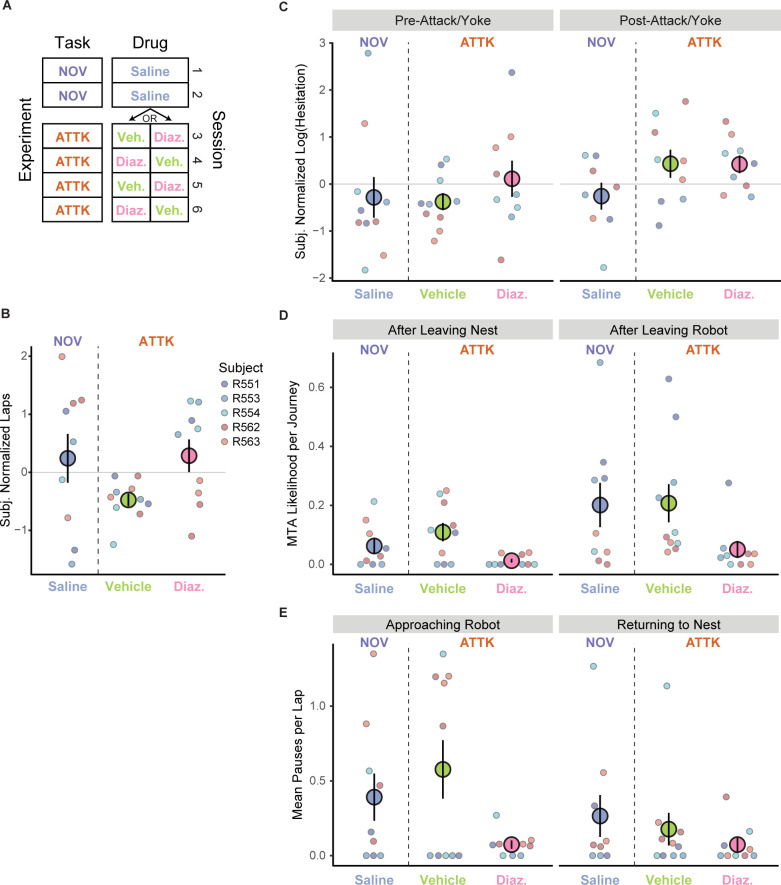
The effects of diazepam on behavior. (**A**) This experiment consisted of 6 sessions: 2 Novelty (NOV), with the robot present, but not attacking, before which the animal received saline injections, and 4 Attack (ATTK) sessions, before which the animal received alternating diazepam or vehicle (Tween-20) injections, with half of the rats starting with vehicle and the other half with diazepam. (**B**) The effect of diazepam and threat on the number of laps that the rats ran. (**C**) The effect of diazepam and threat on hesitation before and after the rat was attacked. (**D**) The effect of diazepam and threat on the probability of the rats engaging in an MTA when moving away from the nest (outbound) and away from the robot (inbound). (**E**) The effect of diazepam and threat on the number of pauses while approaching the robot. Underlying data and code can be found at https://osf.io/v6jt9/. MTA, midtrack abort.

Diazepam is a well-studied and frequently prescribed anxiolytic. Previous work has found that diazepam mediates behavioral measures of anxiety on this task, including changes in hesitation time and number of midtrack aborts [39]. With a separate cohort of rats (*n* = 5, 3 M, 2 F), we administered diazepam or vehicle (Tween-20, see Methods) intraperitoneally (IP) to rats on alternating Attack sessions (Fig 7A). Given diazepam’s half-life of about an hour in rats [83], diazepam likely maintained efficacy during the course of the 1 h session, but was unlikely to affect performance during the following session.

These rats were first trained to find food at alternate locations (nest and far feeder) as with the previous experiment, and then were given 2 days of saline injections to get them used to receiving IP injections and while they continued to run the linear track each day. The robot was present, but not attacking on these 2 saline days, making them Novelty sessions. The rats then went straight to the Attack sessions, alternating diazepam or vehicle, counterbalanced across rats. Single shank silicon probes were implanted unilaterally into CA1 before the first Saline session. Unfortunately, these probes had higher impedances and, thus, did not permit single cell identification but did allow us to assess the LFP.

Diazepam had similar behavioral effects to that seen in previous work [39] and was consistent with the expected effects of an anxiolytic. Specifically, we observed that when administered diazepam on Attack sessions, the rats ran more laps than the Tween-20 controls (Fig 7B; *t*(13.3) = 2.79, *p* = 0.02), were less likely to engage in a midtrack aborts (Fig 7D), and paused less frequently on the track (Fig 7E).

Diazepam did not affect the increase in hesitation seen after being attacked (Fig 7C). We detected a significant interaction in hesitation time between the saline, diazepam, and tween manipulations, and the time on the track (whether before or after the attack or yoked matched-lap), *F*(2, 1335.4) = 8.01, *p* < 0.001, and main effects of the manipulation, *F*(2, 1335.5) = 75.73, *p* < 0.0001, and before or after that marked lap, *F*(1, 1336.0) = 18.17, *p* < 0.0001. During the attack sessions (i.e., administered vehicle or diazepam), the rats showed more hesitation than the non-attack (i.e., saline) sessions in the pre-attack and post-attack periods (|*t*|s > 3.76, ps < 0.001). As a reaction to the first attack, the rats showed more hesitation regardless of whether they were administered diazepam *t*(1336) = 3.75, *p* < 0.001 or the vehicle t(1335) = 3.62, *p* < 0.001. On the non-attack saline sessions, there was no change in hesitation around the matched time, *t*(1336) = 0.59, *p* = 0.55.

However, once the rats entered the track, they were less likely to perform midtrack aborts under diazepam (Fig 7D). We detected main effects of the manipulation, χ^2^(2) = 49.47, *p* < 0.0001, and direction of travel on the track, χ^2^(1) = 20.16, *p* < 0.0001, but did not detect a significant interaction of manipulation with the direction of travel on midtrack aborts, χ^2^(2) = 3.24, *p* = 0.20. The rats made fewer midtrack aborts when administered diazepam than when administered vehicle, *z* = −6.67, *p* < 0.0001, or saline, *z* = −4.83, *p* < 0.0001. As a confirmation of the effects of attacks on behavior, the rats made more MTAs during the vehicle sessions (i.e., when being attacked but not under the influence of anxiolytic) than during saline sessions, *z* = 3.51, *p* < 0.01. As with the first cohort (Fig 5A), rats were also more likely to engage in MTAs when returning to the nest than when moving towards the robot, *z* = 4.49, *p* < 0.0001.

Similar to how diazepam affected midtrack aborts, it also reduced the number of pauses on the track (Fig 7E). We detected a significant interaction of manipulation with the direction of travel for on-track pauses, χ^2^(2) = 8.35, *p* = 0.02, and main effects of manipulation, χ^2^(2) = 95.28, *p* < 0.0001, and direction of travel on the track, χ^2^(1) = 8.16, *p* < 0.01. When moving towards the robot and having been administered diazepam, the rats paused significantly less frequently than when administered vehicle, *z* = −9.19, *p* < 0.0001, or saline, *z* = −7.51, *p* < 0.0001. This was also the case when the rats returned to the nest (versus vehicle: *z* = −4.06, *p* < 0.0001; versus saline: *z* = −5.08, *p* < 0.0001). As with the earlier experiment, the rats paused more often during the vehicle-only attack sessions than during the saline no-attack sessions as they approached the robot, *z* = 3.50, *p* < 0.01, and we did not detect this difference on the return journey, *z* = −1.08, *p* = 0.53.

#### Diazepam reduced SWRs and changed theta asymmetry

In addition to its behavioral effects on anxiety-like behaviors (hesitation in the nest, midtrack aborts, and on-track pausing), diazepam had profound effects on the hippocampal processing in both theta and LIA states.

**Fig 8:**
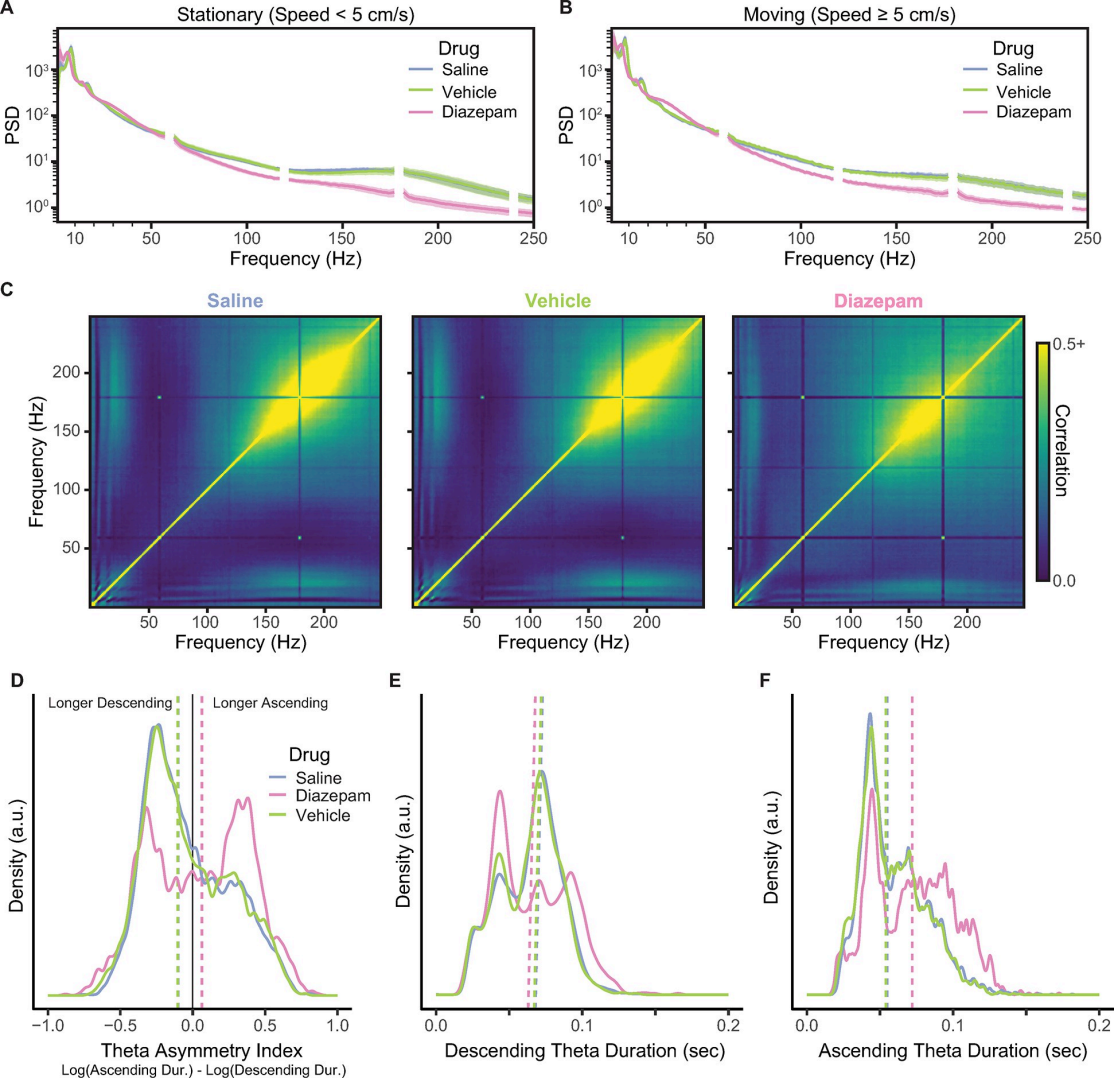
The effects of diazepam on LFPs. (**A**) The effect of diazepam on the PSD when the rats were stationary (speed <5 cm/s). Alternating current at 60 Hz and its harmonics are omitted for this plot. (**B**) The effect of diazepam on the PSD when the rats were moving (speed ≥5 cm/s). (**C**) The reduction in the cross-frequency correlation of frequencies; note the reduced correlation block indicated a reduction in SWR events (150–250 Hz) when the rats were administered diazepam. (**D**) The change in theta asymmetry with the administration of diazepam. The vertical dashed lines indicate the median value. (**E**) How diazepam affects the duration of the descending phase of theta. (**F**) How diazepam affects the duration of the ascending phase of theta. Underlying data and code can be found at https://osf.io/v6jt9/. LFP, local field potential; PSD, power spectral density; SWR, sharp-wave ripple.

As can be seen in Fig 8A and 8B, under the influence of diazepam, SWR oscillatory power (the PSD bump between 150 and 250 Hz) was severely reduced. Because SWRs are transient events, small numbers of these events can be missed in power spectral densities; however, such events appear as a strong block of correlation in cross-frequency correlation plots [84]. Typically, SWR activity can be observed as cross-frequency correlations in the 150 to 250 Hz range, which can be seen when the rats were administered saline or vehicle (Fig 8C). In contrast, when the rats were administered diazepam, these cross-frequency correlations were severely attenuated across the SWR power frequency range of 150 to 250 Hz.

LFPs under diazepam (Fig 8A) showed strong theta and beta power with an increase in 30 to 50 Hz gamma power while the rats were moving (Fig 8B), which likely implies changes in the balance between entorhinal and CA3 inputs [85,86]. Importantly, the peak frequency of theta shifted discernibly from 8 to 6 Hz under the influence of diazepam.

Diazepam significantly altered theta asymmetry during the outbound journeys (Fig 8D) by making the ascending periods of theta longer and the descending periods briefer. When the rats were administered diazepam, they had significantly greater theta asymmetry (via Kolmogorov–Smirnov test: diazepam versus saline: *D* = 0.17, *p* < 0.0001; diazepam versus vehicle: *D* = 0.16, *p* < 0.0001). There was no difference in theta asymmetry when the rats were administered vehicle versus saline, *D* = 0.02, *p* = 0.64. The duration of the descending phase of theta (Fig 8E) was shorter when administered diazepam (diazepam versus saline: *D* = 0.12, *p* < 0.0001; diazepam versus vehicle: *D* = 0.14, *p* < 0.0001), and there was no detected difference in the duration of the descending phase between saline and vehicle (*D* = 0.03, *p* = 0.12). The duration of the ascending phase (Fig 8F) was longer when administered diazepam (diazepam versus saline: *D* = 0.23, *p* < 0.0001; diazepam versus tween: *D* = 0.24, *p* < 0.0001), and there was no detected difference when they were administered saline and vehicle, *D* = 0.03, *p* = 0.10. This pattern of results suggests that diazepam profoundly affected theta symmetry by reducing the descending duration and increasing the ascending which inverts theta asymmetry.

## Discussion

Rats facing approach-avoidance conflict on the predator-inhabited foraging task (the “robogator” task) showed behavioral changes, consistent both with previous studies and with a hypothesis that rats were showing increased concern about being attacked by the robot (worry). There was increased time spent hesitating in the nest, which led to a decrease in the number of laps run, and, hence, a decrease in the food rewards received. Additionally, we observed an increase in the rate of midtrack aborts, in which rats ran out into the main track, but fled back to the nest before reaching the goal, and increased on-track pausing, in which rats paused on the track for a few seconds before proceeding.

Importantly, the hippocampal representations were fundamentally different during each of the 3 observed behaviors. During hesitation, decoding hippocampal activity was more likely to reveal the robot’s location and less likely the food goal location during HSEs (hippocampal cell bursts) after the rats had been attacked. Prior to engaging in an MTA, the decoded posteriors were more likely to include the attack threshold and the robot location, than in matched lap controls. The hippocampus remained in the theta state through the MTA event, and the hippocampal activity generally represented the rats’ planned direction of travel; however, after turning back towards the nest, hippocampal activity in the ascending theta phases (that usually represent positions ahead of the animal on approach tasks) decoded to the robot location and the attack threshold even though they were behind the animal. During on-track pausing, decodings during this portion of theta cycles included both the robot and the far feeder locations, suggesting pauses may arise from conflict between the danger to avoid and the goal to approach.

Transient representations of negatively valued potential destinations during hippocampal SWRs have been seen on a novel shock-task [36], but this was on a novel maze, on which hippocampal activity during SWRs is known to be different [66,67], and it was not known what caused these hippocampal transient representations of the danger zone. As noted above, we also found increased decoding to the robot’s location during HSEs during hesitation in the nest. Fascinatingly, these observed increases in decoding of the robot’s location did not arise from changes in the sets of cells participating in the HSEs, but rather from a change in the spatial tuning of the hippocampal cells after the rat was attacked. When we used the post-attack tuning to decode hippocampal activity during HSEs in the nest, we found an increased decoding to the robot’s location, even with HSEs from before the attack. Conversely, there was no change in the decoding to the robot location during HSEs after the attack if we used the pre-attack tuning as the training set for decoding. This remarkable result provides a fascinating mechanism for increased worry through hippocampal processes—random bursts of cell activity during HSEs and SWRs would pull an increased representation of the robot, not because there was attention to the robot during the HSEs and SWRs, but because the place cells had adjusted to include more robot-related fields.

One of the most interesting discoveries revealed in these data were the changes in spatial tuning after the first time the rat was attacked. As can be seen in Fig 3C and 3D, after being attacked during Session 5 (the first Attack session), the spatial tuning increased at the location of the robot and the area beyond the attack threshold. Importantly, however, these new fields were not due to the novelty of the robot itself, as the robot was present during the Novelty sessions (Sessions 3 and 4), but the spatial tuning did not change until the rat was attacked on Session 5.

Place fields have been observed to change after important episodic events, such as maze changes, attention-grabbing events such as sudden environmental manipulations, and the presence of novel objects (see [48] for review). Place fields have been seen to appear when novel objects are placed on the track [87–89], through direct induction of synaptic plasticity [90,91], or when rats stop and head-scan, presumably attending to distal external cues [74]. In fear-conditioning experiments, novel place fields have been seen to appear at the location of the animal after the first shock [72,73]. Importantly, however, all of these changes occurred at the location of the rat, presumably reflecting the rat’s personal contextual information at the time of the episodic event. Place field changes on the robogator task have been previously reported as being more likely to occur near the robot than near the nest [2,46], but the time course of those changes was unknown. Here, we observed the sudden appearance of new place fields and an increase in existing spatial activity, not at the location of the rat at the moment of attack, but rather at the location of the robot and space beyond the attack threshold—a new episodic memory of the object of worry.

During MTAs, the hippocampus remained in the theta state. Consistent with approach tasks, the hippocampus decoded to where the animal was during the descending phase of theta, and future paths during the ascending phase of theta [24,25,27,53–55]. During the MTA, just before the animal turned around, the posterior probability in the ascending phase of the theta cycle concentrated at the attack threshold, the point where the robot might attack the rat, making this the effective next choice point. As the animal turned, the posterior probabilities during the ascending phase of theta started to break apart, but contained increased information about that attack threshold, even as the animal started to flee back to the nest. Previous work has found that theta sequences can proceed in a different direction from the orientation of the animal, but in these previous studies, theta sequences have always been in the actual direction of motion [92,93]. These data suggest, instead, that cell firing during the ascending phase of theta was representing attentional locations that the animal was concerned about, consistent with the transient jumps to the danger zone during the second phase of theta seen on the active place avoidance task [56].

In a classical fear-conditioning (cued-shock) task, Gruene and colleagues [94] identified a novel behavioral response to fear-inducing stimuli in which some rats (particularly female rats) suddenly darted to the other side of the small enclosure, even though they could not escape the oncoming shock. It is an interesting question whether midtrack aborts are related to this darting behavior. One major difference between midtrack aborts and darting behaviors is that midtrack aborts are directed towards a point of safety and indicate a switch in the animal’s goal (i.e., from food to safety) and it is currently unknown what the function of darting is. The extent to which these behaviors could be mediated by similar underlying neural mechanisms is unknown and will have to be investigated in future experiments. Gruene and colleagues [94] found that there were sex differences in freezing and darting behavior in response to a learned inescapable shock-cue pairing. We lacked the statistical power to perform rigorous statistical analysis of sex differences. Thus, we could not evaluate whether there was a true difference in the behavior of the sexes on this task; a much larger sample would be required to detect any differences.

The hippocampus remained in relatively normal theta during the on-track pauses, and the spatial decoding during the 2 phases of theta were relatively normal, although there was increased decoding to the robot and feeder locations during the pause. It is likely that these on-track pauses were initiated by other decision systems, not involving the hippocampus, but that information about the threat and reward was represented during the pause. To what extent on-track pauses relate to freezing seen during active fear [18,95–98] or whether they relate to tonic immobility [99,100] remains unclear and will likely require further study.

Fascinatingly, diazepam affected the hippocampal processes associated with the anxiety-like behaviors, in line with its effects on anxiety-like behavior. We found that diazepam dramatically reduced the number of midtrack aborts and on-track pausing behaviors. In line with these behavioral reductions, diazepam affected the underlying hippocampal processes associated with each of these. The effects of benzodiazepines on the hippocampus are relatively well studied, but not as animals are behaving on a decision-making or anxiogenic task. For example, rodents allowed to move in an open field or walking on a forced-walk treadmill show a downward shift in theta frequency under diazepam [101–103]. Additionally, diazepam is known to interfere with SWR events [104–106], but it is not known whether these changes occur during anxiety-related behaviors. Consistent with this, we found that diazepam reduced the central frequencies of theta and beta oscillations and severely reduced SWR power in the hippocampus (Fig 8). An interesting effect of diazepam was that it reversed theta asymmetry, which could dramatically impact how the hippocampus represents spatial information.

Because diazepam was delivered systemically, these effects could be due to primary effects of diazepam on hippocampal neurophysiology or to other systems impinging on hippocampus either directly or indirectly. However, these data raise the fascinating possibility that one mechanism through which diazepam achieves its anxiolytic effects is by interfering with hippocampal functionality and the ability to represent objects of worry.

Ventral hippocampus and amygdala are more often ascribed roles in threat recognition and avoidance [99,107–110]. Previous work has found that the amygdala, in particular, is involved in variants of the robogator task [37,38,111]. The results presented here have identified that the dorsal hippocampus changes spatial tuning in the light of novel threats (Fig 3) and encodes that threat during avoidance (Fig 5). How well dorsal and ventral hippocampus encode coordinated versus independent information about threats, particularly novel threats, remains an open question [111–115], as does how these representations impact amygdala and other decision system components.

Theories have long hypothesized that anxiety involves imagination, and worry involves negative episodic thinking [1–5]. Extensive data has identified a role for the hippocampus in the imagination of other places and other times [6,7,10,48], and, in particular in planning, particularly about positive futures to approach [9,24,25]. Here, we find that these same hippocampal processes also encode important information about negative events, both experienced dangers and potentially dangerous futures.

## Methods

### Subjects

A total of 11 (6 male, 5 female) Brown Norway rats aged between 7 and 10 months served as experimental subjects. Six (3 male, 3 female) rats were subjects in the neural ensemble recording experiment, and the remaining 5 (3 male, 2 female) rats were subjects in the diazepam experiment. All rats were maintained on a 14:10 h light/dark cycle. During the experiment, rats were food restricted such that their entire daily complement of food was earned from 45 mg food pellets (full-nutrition, Test Diet) in the foraging arena. Rats were always kept above 80% free-feeding weights and had unlimited access to water when in their home cages. All procedures were approved by the University of Minnesota (UMN) Institutional Animal Care and Use Committee (IACUC) under protocol numbers 1610-34226A, 1910-37469A, and 2207-40198A, and were performed in accordance with NIH guidelines.

The 2 cohorts of rats had to meet different criteria prior to the surgery. The first cohort of rats, which did not receive the drug manipulation, was trained to run a minimum of 50 laps on the linear track for 2 consecutive days. The second cohort met a duration criteria of being trained on the linear track for 7 days prior to the surgery. In both cases, the rats had ad libitum access to food (Teklad pellets) for at least 3 days prior to surgery.

### Surgery

Prior to the surgery, we anesthetized the rat with 0.5% to 2% isoflurane mixed with medical-grade O_2_ in an induction chamber and maintained this level of anesthesia throughout the surgery via a Somnosuite system (Kent Scientific, Torrington, Connecticut, United States of America). Once the rat was anesthetized, we placed the rat on a stereotax (Kopf Instruments, Tujunga, California, USA). We removed the skull and dura above the dorsal hippocampus, chronically implanted silicon probes anterior-posterior at −3.8 mm and medial-lateral +2.5 mm or −2.5 mm (probe details in the next paragraph), affixed the probe and amplifier assembly by its connector to the skull surface with MetaBond (Parkell, Edgewood, New York, USA), sealed the surgical hole with wax, and surrounded the assembly with a protective shroud that was printed on a 3D printer (Formlabs, Somerville, Massachusetts). All probes had one dimension of vertical movement and were moved to successfully reach CA1 as identified by SWR reversals with the exception of the left-hemispheric probe in R643, which was omitted from analysis. After the surgery, the rats recovered in an incubator to maintain body temperature and were orally given 0.8 ml of Children’s Tylenol to alleviate discomfort. During the surgery and for the 3 days following, the rats were administered 25 mg/kg of Baytril and 5 mg/kg of carprofen. During the post-surgery days, we cleaned the surgical site with betadine. We resumed behavioral training with the rat after 3 days.

The silicon probes used varied by the experiment. With the first cohort of rats that served for the neural ensemble recordings (3 male, 3 female), we bilaterally implanted 64-channel, 4-shank Cambridge Neurotech P-1 probes into the dorsal hippocampus. With most of the diazepam cohort, we unilaterally implanted 64-channel, single-shank Cambridge Neurotech H-3 silicon probes into the left hemisphere. One male rat in the diazepam cohort was unilaterally implanted into the left hemisphere with a 32-channel Cambridge, 2-shank Neurotech E-2 silicon probe. The silicon probes used in the pharmacological experiment had been replated after being used in a prior experiment, but this did not result in good enough conductance to identify cells.

### Diazepam

A cohort of 5 rats were administered an anxiolytic drug manipulation of diazepam. Diazepam (2 mg/kg) was dissolved in Tween-20 to prepare a stock solution, which we then diluted with 0.9% saline. For our control manipulation, we used a vehicle solution (10% Tween-20 in saline). Both diazepam and Tween-20 were sourced from Sigma Adlrich (St. Louis, Missouri). We chose this dose for diazepam due to its known efficacy in behaving rats [116] and previous behavioral experiments on this task [39]. We intraperitoneally administered all injections 5 min prior to each session. During the experiment, we alternated the administration of diazepam with the vehicle solution within each subject, daily. Diazepam was only administered once per day prior to the experiment. Given diazepam’s half-life (and its major psychoactive metabolite desmethyldiazepam) of about an hour in rats [83], diazepam likely maintained efficacy during the course of the 1 h session and was unlikely to affect performance during the following session.

In one diazepam session in one rat (an early attack session of R551), the animal was accidentally administered a doubly diluted dose of diazepam (i.e., 0.2 mg/kg dose). For consistency with the rest of the experiment, we removed the data from this session from analyses.

### Statistical and computing resources

Data collection and analyses were conducted via a combination of MATLAB, Python, and R scripts. MATLAB (version R2015b; MathWorks, Natick, Massachusetts, USA) was used to record behavioral data, manage the task, generate robot attacks, and deliver food. Python (version 3.9.4) code was used to perform data management, signal analysis, identification of behavioral events, and neural decoding. Notable Python packages that were utilized were *neuroDSP* (version 2.1.0), *numpy* (version 1.24.3), *scipy* (version 1.10.1), *pandas* (version 1.5.3), and *matplotlib* (version 3.6.2). R (version 4.2.2) was used to perform statistical analyses and plotting in RStudio. Notable R packages that were used were *car* (version 3.1-1), *dplyr* (version 1.0.10), *emmeans* (version 1.8.4-1), *forcats* (version 0.5.2), *ggplot2* (version 3.4.0), *lme4* (version 1.1-31), *performance* (version 0.10.2), and *tibble* (version 3.1.8).

### Statistical analysis techniques

Unless otherwise specified, the *p*-values presented in the Results are adjusted *p*-values and can be directly compared to the alpha of 0.05.

Statistical analyses used a mixed-model framework by including a random effect of the subject within generalized linearized mixed effects regressions via the *lme4* R package. These mixed-effects models provide the appropriate mix of power given within-subject effects [117]. The number of laps, SWR rates, and HSE rates were modeled as a Gaussian distribution. Since hesitation duration was non-Gaussian, we log-normalized it prior to fitting a linear mixed effects regression. HSE decodings were modeled as binomial distributions after coercing values less than 0.5 to 0 and values greater than 0.5 to 1. The likelihood of a midtrack abort on an outbound journey was also modeled as a binomial distribution. The number of pauses on a journey was modeled as a zero-inflated Poisson distribution. For significant interactions and main effects, we used the *emmeans* R package to determine significant differences. This analysis adjusted the degrees of freedom via the Kenward–Roger method and the *p*-values via the Tukey method. To compare distributions of multimodal data (e.g., theta asymmetry), we utilized the Kolmogorov–Smirnov test in R and adjusted *p*-values via the Benjamini and Hochberg method to account for multiple comparisons.

To statistically assess changes in power spectral densities, we used dependent *t* tests via the Python *scipy*.*stats*.*ttest_rel* function. Power of a frequency band was calculated from the average power across the frequency of its range. When we assessed the differences in spectral power at 2 time points, we compared the delta (1 to 4 Hz), theta (6 to 10 Hz), beta (15 to 20 Hz), and SWR (120 to 250 Hz) frequency bands. Since we were making multiple assessments across the PSD, we used a Sidak correction to adjust statistical significance (alpha) values.

### Arena

The arena was 111 cm in length and consisted of a nest, a track, and a robot bay on the side that formed an “L” shape that was constructed with DUPLO bricks (LEGO, Billund, Denmark). The DUPLO bricks were placed in a random and irregular color scheme to provide identifiable patterns to facilitate the formation of place cells. The nest was 19-cm long and 26-cm wide with partial walls creating a smaller opening that connected it to the track. The track was 67-cm long and 26-cm wide with the robot bay on the side at the far end from the nest. The robot bay was 22 cm along the side shared with the track and 35-cm deep. The robot was placed at an angle in the robot bay such that it would move towards the rat when it attacked. On sessions that the robot was not presented, a wall made of DUPLO bricks blocked off the robot bay.

Two feeders (Med-Associates, Georgia, Vermont) were located on opposite sides of the track with one centered in the nest and one at the far end of the linear track. The latter feeder was on the opposite side of the robot, which meant that the rat had to pass by the robot to reach the delivered food pellet. The feeders provided food pellets when the rat approached it, but only if the rat had gone to the other feeder since the last visit. Two pellets were provided at each feeder to all rats for all sessions in the experiment that did not have a drug manipulation. The experiment with the drug manipulation had the feeders deliver 2 pellets to the female rats and 3 pellets to the male rats. We used different pellet counts in the drug manipulation experiment to better control for the number of laps that needed to be run to achieve satiation across the males and females.

There were 3 session types across the experimental sessions: Linear Track, Novelty, and Attack. On Linear Track sessions, the robot bay was walled off, which left the purely linear maze for the rat to run back and forth on. On Novelty sessions, the robot bay was open with the robot present but it did not engage in any actions during the session. On Attack sessions, the robot attack was triggered by the rat crossing an unmarked threshold. The first attack on these sessions could not occur until after the 15th lap. On this and subsequent laps, there was a 20% chance of the robot attacking when the rat crossed the threshold.

### Robot

We used a robot constructed from the SPIK3R set (set number 31313, LEGO Mindstorms, Billund, Denmark), which has a scorpion-like shape and we modified the design for our purposes. The primary modification was that we removed the legs from that design and made it run on wheels along a grooved track. The robot was controlled by in-house programming (MATLAB), which used the position of the animal as a trigger to initiate an “attack” behavior by the robot. When an attack was initiated, the robot would make a screeching noise for 1 s before surging forward and moving its arms. After the attack, the robot would move back to its starting position.

### Behavioral quantification

We tracked the position of the rat by identifying the location of a red LED that was attached to the headstage. We tracked the LED by finding the position of maximum brightness within the confines of the foraging arena. If the position of maximum brightness was not above a value threshold, then we noted that we had lost track of the rat. The rat’s position was recorded at a rate of 30 Hz. From the change in the rat’s position, we identified a number of important behavioral events. When we normalized the data by subject, we subtracted the mean and divided by the standard deviation of that subject’s values.

#### Hesitation

Hesitation was identified as anytime the rat was inside the nest or in the doorway to the nest. Hesitation time started when the rat entered the nest space from the track and ended when the rat left the doorway, proceeding out onto the track. We omitted the amount of time that the rat spent within 2 cm of the feeder’s location.

#### MidTrack aborts (MTAs)

We identified candidate MTAs as instances where the rat entered the track and returned to the door. As the minimal criteria, the rat’s headstage LED had to be on the track for 0.5 s and to have traveled 5 cm on the track. When we required a precise time for identification of the MTA itself, we visually assessed the candidate MTAs and removed instances when the animal did not at least turn their body to be perpendicular to the length of the track. Cases where a candidate MTA was identified but the animal did not turn typically indicated instances of the rat stretching onto the track without fully leaving the door zone, which resulted in them backing up into the nest. Given the qualitatively different nature of this action, we chose to exclude instances of it for MTA analysis. We identified the time of the MTA as when the rat’s head began to move in the direction of the eventual turn as this was the most consistent time point for analysis. Midtrack aborts were also identified when the animal was returning to the nest. In these cases, we used the same criteria as the outbound journey, but did not go back to assess the exact time when engaging in the turn.

#### Pauses

We identified pause events from the behavioral tracking. Pauses were instances that the rat stopped moving for a prolonged period of time. Since the LED was attached to the rat’s headstage, we ran a 2 Hz low pass filter to remove sharp movements. From this smoothed movement, we identified instances when the animal moved by less than 1 cm along the long axis of the track for over half a second. The start and end of each pause were identified as when the animal began and stopped meeting this criteria, respectively.

#### Pre and post attack yoking across sessions

The first time that a rat is attacked by the robot during a session likely indicates a marked change in the rat’s behavior and, likely, a change in their neural representation of the environment. However, even during non-attack sessions the rat’s behavior changed as they engaged with the maze and we, thus, needed a valid experiential match across sessions for a good comparison. To do this, we matched the number of laps each animal ran before being attacked on the Attack sessions to the Linear Track and Novelty sessions. For each Attack session, we identified the first lap that the rat was attacked by the robot. This resulted in 4 lap counts that identified the number of laps the rat was able to run on the Attack sessions prior to being attacked (e.g., during sessions 5 to 8, R646 was attacked on laps 15, 19, 16, 18, respectively). For each rat, we randomly shuffled these lap counts and assigned them to the Linear Track and Novelty sessions (thus, for R646, we used 16 and 19 for the Linear Track sessions and 18 and 15 for the Novelty sessions). From these matched lap indices, we identified the time prior to (Pre) and after (Post) that lap index as the match comparison for the Attack sessions. Thus, the transition between pre and post were matched across the different session types, and we could compare the behavior before (pre) and after (post) the lap count on Linear Track and Novelty sessions with when before (pre) and after (post) an attack would occur on the Attack sessions. This procedure thus resulted in comparable amounts of maze experience, in both the mean and variance, for each animal when we performed comparisons across sessions.

For simplicity, we refer to analyses that compare early (before first attack lap or yoked lap) to late (after first attack lap or yoked lap) as “pre vs. post” (pre/post) analyses, with pre being the early condition and post the late.

### Cell identification

Recordings were taken using an Intan RHD 2000 system (Intan, Los Angeles, California, USA) at 30 kHz. With the first cohort’s data, a band-stop filter at 60 Hz was implemented at the time of recording. Voltage signals were first normalized by subtracting the median signal at each sample across the duration of the recording for common noise rejection. We then used Kilosort 2.0 [118] to sort the cellular activity into putative cells and Phy [119] to visually assess the quality of the clusters and merge and split them as necessary. Following the spike-sorting process, we cross-correlated each putative cell’s spike waveforms with the median amplitude spike to determine the optimal temporal spike alignment and adjusted the spike timings appropriately. Putative pyramidal cells were identified as those cells with a mean firing rate less than 10 spikes/sec and a peak to valley duration ratio of greater than 0.4.

We identified the locations of the probes for both experiments post sacrifice by applying cresyl violet to coronal slices of the brain.

### Theta phase identification

To differentiate the ascending and descending phases of theta, we utilized a cycle-by-cycle technique [120]. We filtered the LFP to specific and broad theta band frequencies of 6 to 10 Hz and 6 to 40 Hz, respectively. From the specific signal, we identified the simple peaks and troughs as the maxima and minima between 0 power crossings. From these points, we then looked at the broader LFP, which better describes the saw-tooth pattern of theta in the hippocampus and identified more accurate time points of the peak and trough.

### Power spectral density analyses

For analyses of the LFP’s PSD, we used the spectrogram function from the *scipy* package, with a bin length of 1 s. When the PSD analysis needed to be aligned with an event (e.g., pauses and midtrack aborts), we used the spectrogram function to define custom time bins for the analysis around the event. Given that these events were typically briefer, we used a bin length of 0.25 s. After computing the spectrograms, we used Welch’s method to determine the PSD for the separately identified before and after event time periods. When comparing power spectral densities, we defined the frequency bins using standard frequencies. Delta was defined as 1 to 4 Hz, theta as 6 to 10 Hz, beta as 15 to 20 Hz, and gamma as 30 to 80 Hz. SWRs were defined as the more narrow 150 to 250 for identifying individual events, but a broader 120 to 250 Hz for PSD analysis. We used the wider band for many analyses because we observed that the cross frequency correlations that typically indicate the sharp-wave frequency band began at that point with the rats we were working with (see, for example, Fig 4A).

### Sharp-wave ripple (SWR) event identification

To identify SWRs, we first found the channel that had the greatest SWR oscillatory power in the 120 to 250 Hz range above and beyond the aperiodic activity. We identified this channel for each probe shank by calculating the PSD of each channel on the shank, removing the aperiodic component, and then comparing the residual power within the power spectrum. We identified the aperiodic component by fitting a robust linear regression to the log-log PSD in the 80 to 400 Hz frequency range. This approach was taken because the log-log PSD linearizes the aperiodic and we used a robust linear regression so that the influence of the oscillatory SWR power on the regression was minimized. After identifying the aperiodic component, we removed it from the PSD that gave us the remainder, which was primarily the SWR power. The best candidate SWR layer was identified as the channel on the shank with the largest SWR power in the 120 to 250 Hz frequency range. We qualitatively validated the best SWR layer by visually examining the LFPs after bandpass filtering it between 120 and 250 Hz, ensuring that the identified channel had good SWRs.

SWR events were defined as instances when the LFP power in the 150 to 250 Hz range was more than 4 standard deviations above its mean for more than 20 ms. To determine when this criteria was met, we bandpass filtered the LFP and applied a Hilbert transform to shift it into the complex number domain. We normalized the power and then identified candidate SWR events by determining when the power was above the criteria threshold. We merged candidate instances when the gap between the end and start was less than 5 ms or when the gap between the start of 2 instances was less than 20 ms. After this merge, we filtered these candidates to just those instances that lasted longer than 20 ms.

### High-synchrony event (HSE) identification

We identified HSE by identifying bursts of pyramidal cell activity. We summed the spiking of putative pyramidal cells in 1 ms bins. Next, we applied a Gaussian kernel with a standard deviation of 7 ms to these bins. From this data, we identified candidate HSEs as those instances when the kerneled spike activity was more than 3 standard deviations above the mean over the session. From this set of candidates, we only kept instances during which the higher spike activity was maintained for more than 20 ms and less than 750 ms with the starts and ends of these instances being when they started and stopped meeting the greater than 3 standard deviations criteria.

### Changes in spatial tuning

When examining the changes in spatial tuning, we segmented the track into 64 bins along the length of the track and 3 bins for the direction of travel (moving towards the robot, stationary, moving towards the nest). When constructing cellular tuning curves on these dimensions, we only used cellular activity while the hippocampal LFP was dominated by theta and only during its descending phase. This approach should minimize the influence of cellular activity during sharp wave ripples and theta sequences, and thus maximize the local spatial accuracy. The criterion for the hippocampus being dominated by theta was when the within-subject-normalized log ratio of theta and delta power (i.e., log(power_theta_/power_delta_)) was ≥0.5. From the cellular activity during these periods, we constructed the tuning curve, and then normalized it by the rats’ occupancy in the space while traveling in the specified direction. For statistical comparisons, we collapsed over the length of spatial bins that comprised each zone.

### Decoding

We used a one-step Bayesian approach [121] to decode the spatial information that is most likely represented by ensemble neural activity in the dorsal hippocampus. To estimate the representation during a period of time, this approach assumes that the firing rate of neurons follows a Poisson distribution and that the activity of the neurons are independent.

To decode the posterior probability from the ensemble of neural activity requires 3 steps: (1) create tuning curves of the neurons; (2) create a temporal spike matrix of the neural activity; and (3) calculate the predicted posterior probability. We first constructed tuning curves (as described in Changes in Spatial Tuning), which resulted in segmentation of each cell’s activity into 192 spiking-activity bins (64 spatial segments along the length of the track by the 3 bins for the direction of movement—moving towards the nest, moving away from the nest, stationary). This segmentation effectively treats the approach and return from the robot as separate track segments. Secondly, depending on the analysis, we used different temporal spike matrices. When comparing differences over the ascending and descending phases of theta, we counted each cell’s activity during the phases of theta over the experiment (see Theta-Phase Identification Methods section for details). When examining HSEs, we counted each cell’s activity during the entire duration of the HSEs. Thirdly, we estimate the posterior probability by applying Bayes theorem to each location

$$P(x_{t}|n_{t})=P(n_{t}|x_{t})\times P(x)÷P(n),$$

which can be calculated via

$$P(x_{t}|n_{t})=\prod_{i=1}^{N}{A_{i}(x)}^{n_{i,t}}\times \frac{1}{X}{÷e}^{\tau \sum_{i=1}^{N}A_{i}(x)}.$$

*P*(*x_t_*|*n_t_*) is the conditional probability of the location, *x*, given the neural ensembles’ number of spikes, *n*, at time, *t*. The first term is the conditional probability of the observed number of spikes in the neural ensemble, 1 through N, given each neuron’s expected activity, *A_i_*, at that location. The expected activity of the cells, *A_i_*, at the location is given by the occupancy-normalized tuning curve. The second term is the probability of the location being occupied given uniform occupancy over the maze, which is given by 1 divided by the number of locations, X. The final term is the predicted spiking, which is given by the predicted spiking activity at *x* times the duration, τ. After *P*(*x_t_*|*n_t_*) is calculated for all of the locations at time *t*, it is normalized by dividing it by the sum of the estimated probabilities across all locations at time *t* (i.e., ∑*P*(*x_j,t_*|*n_t_*) = 1).

In any given session, we only applied this decoding operation if there were at least 10 putative neurons during the session.

## Supporting information

S1 Fig Change in path due to attack. The paths taken during outbound laps by each rat. Thin black lines are individual journeys (transparency adjusted by the number of journeys, less emphasis on each journey when there are more journeys). The borders of the maze and feeder thresholds (i.e., the lines to cross for the feeder on that end to fire) are shown as thick black lines. The shown paths omit journeys when the animal engaged in a midtrack abort or the animal was attacked. The median path is shown during the approach as the colored lines, which are directly compared in the rightmost column. Prior to being attacked, the rats showed stereotyped approaches, but after being attacked some of the animals changed their approach. However, there was significant heterogeneity in that change with three of the rats (R643, R646, and R650) taking a new route that was further away from the robot, 2 (R645 and R647) not markedly changing, and 1 (R680) moving closer to the robot during the approach before veering back towards the feeder.
(PDF)

## Abbreviations

HSE: high-synchrony event
IACUC: Institutional Animal Care and Use Committee
LFP: local field potential
LIA: large-amplitude irregular activity
MTA: midtrack abort
PSD: power spectral density
SWR: sharp-wave ripple
