# Supplementary material for: Dorsal hippocampus represents locations to avoid as well as locations to approach during approach-avoidance conflict
Source: PLoS Biol. 2025 Jan 14;23(1):e3002954. doi: 10.1371/journal.pbio.3002954 (PMC11731767; doi:10.1371/journal.pbio.3002954)
Supplement: S1 Fig — The paths taken during outbound laps by each rat. Thin black lines are individual journeys (transparency adjusted by the number of journeys, less emphasis on each journey when there are more journeys). The borders of the maze and feeder thresholds (i.e., the lines to cross for the feeder on that end to fire) are shown as thick black lines. The shown paths omit journeys when the animal engaged in a midtrack abort or the animal was attacked. The median path is shown during the approach as the colored lines, which are directly compared in the rightmost column. Prior to being attacked, the rats showed stereotyped approaches, but after being attacked some of the animals changed their approach. However, there was significant heterogeneity in that change with three of the rats (R643, R646, and R650) taking a new route that was further away from the robot, 2 (R645 and R647) not markedly changing, and 1 (R680) moving closer to the robot during the approach before veering back towards the feeder. (PDF) [file pbio.3002954.s001.pdf]

# Dorsal hippocampus represents locations to avoid as well as locations to approach during approach-avoidance conflict

## Supplementary Information

Olivia L. Calvin, Matthew T. Erickson, Cody J. Walters[1], A. David Redish*

Department of Neuroscience
University of Minnesota
Minneapolis MN 55455

[1] Current affiliation: Senior Editor, *Nature Communications*.

* Corresponding author: redish@umn.edu

**Figure S1. Change in Path Due to Attack.** The paths taken during outbound laps by each rat. Thin black lines are individual journeys (transparency adjusted by the number of journeys, less emphasis on each journey when there are more journeys). The borders of the maze and feeder thresholds (i.e., the lines to cross for the feeder on that end to fire) are shown as thick black lines. The shown paths omit journeys when the animal engaged in a midtrack abort or the animal was attacked. The median path is shown during the approach as the colored lines, which are directly compared in the rightmost column. Prior to being attacked the rats showed stereotyped approaches, but after being attacked some of the animals changed their approach. However, there was significant heterogeneity in that change with three of the rats (R643, R646, R650) taking a new route that was further away from the robot, two (R645, R647) not markedly changing, and one (R680) moving closer to the robot during the approach before veering back towards the feeder. Underlying data and code can be found on Open Science Foundation (see data statement for details).

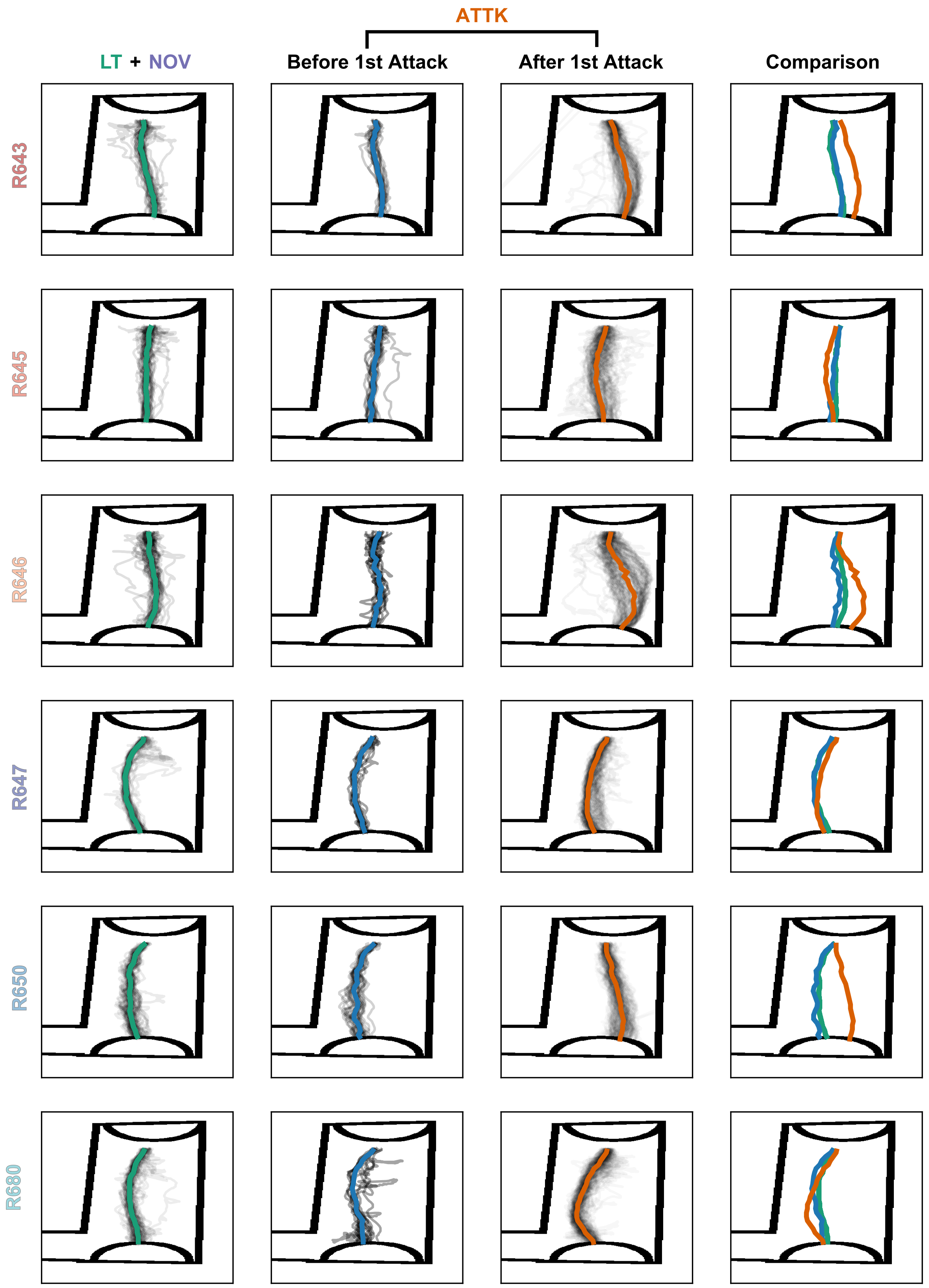
ATTK
LT + NOV
Before 1st Attack
After 1st Attack
Comparison
R643
R645
R646
R647
R650
R680
